# Gossip

**Published:** 1882-02

**Authors:** 


### GOSSIP.

What is gossip but the patent sign of vulgarity in heart and mind? Intellect that never rises beyond the small mean facts of personal history, fluidity which cannot keep to itself what has been told even in confidence, and that goes about swelling mole-hills into mountains, can claim no respect, make out no case for itself against the decree of gross, vile, stupid vulgarity recorded against it. It is as thoroughly vulgar as is curiosity; and between prying into things with which we have no concern, and retailing gossip which is no business of ours to handle at all, there is not a hair's breadth to choose. Each is equally the sign of utter and entire vulgarity; and if one is the bull's-eye the other is the gold. But both are faults to be found growing as rank as weeds by the wayside, and it would be hard to count upon one's fingers among our largest circle of acquaintance those who are absolutely free from the vice of gossip and the vulgarity of curiosity. So, too, intensely vulgar is that habit of talking people over indulged in by some who yet have all the outward seeming of thorough breeding. There are houses where one goes where the rule is to discuss, not always amiably, all those guests, misnamed friends, who have just left after the dinner is over or the soirée has come to an end. Just as there are people who make it a rule to discuss, also by no means amiably, the host and hostess who have this moment entertained them, all the arrangements made in their honor, and all the guests who have been invited to visit them. There is no shame felt by these queer "friends," these now hosts and now guests, because there is no sense of kindness received or hospitality displayed. The thing is only a social bargain, a *quid pro quo* involving no delicacy, because devoid of all sincerity. It is a mere social form like saying "Dear Sir" to a man for whom you have no regard, and signing an insolent letter "Your obedient servant," or a false one "Yours faithfully." So many dinners for so many, and then the account is squared. What never does come quite square is the amount of ill-nature that serves as the *chasse café* when all is done, and the guests fall foul of the hostess and the hostess sneers at the guests. It is only one step lower in the scale than that other act of vulgarity—criticising you to your face—in which so many people indulge. It is not the least well-bred of the two who accepts the annoyance quietly, and forbears to retaliate with the same weapons or different ones. Indeed, quietness and gentleness and the dignity which can bear unprovoked assaults without apparent annoyance, yet without craven submission, count among the very antitheses of vulgarity. Would that we had more of such good breeding in the world! As good grain chokes out the rampant weeds, so would tempers of that kind choke out the vulgar peevishness which "shows a spirit" at every turn, as well as that still more vulgar insolence which provokes it.—*The Queen*

